# Loss of ATF3 exacerbates liver damage through the activation of mTOR/p70S6K/ HIF-1α signaling pathway in liver inflammatory injury

**DOI:** 10.1038/s41419-018-0894-1

**Published:** 2018-09-05

**Authors:** Qiang Zhu, Han Wang, Bin Jiang, Xuhao Ni, Longfeng Jiang, Changyong Li, Xuehao Wang, Feng Zhang, Bibo Ke, Ling Lu

**Affiliations:** 1 Liver Transplantation Center, First Affiliated Hospital, Nanjing Medical University, Nanjing, China; 2 Children’s Hospital of Nanjing Medical University, Nanjing, China; 3 Department of Physiology, School of Basic Medical Sciences, Wuhan University, Wuhan, China; 4 The Dumont-UCLA Transplant Center, Division of Liver and Pancreas Transplantation, Department of Surgery, David Geffen School of Medicine at University of California-Los Angeles, Los Angeles, CA USA

## Abstract

Activating transcription factor 3 (ATF3) is a stress-induced transcription factor that plays important roles in regulating immune and metabolic homeostasis. Activation of the mechanistic target of rapamycin (mTOR) and hypoxia-inducible factor (HIF) transcription factors are crucial for the regulation of immune cell function. Here, we investigated the mechanism by which the ATF3/mTOR/HIF-1 axis regulates immune responses in a liver ischemia/reperfusion injury (IRI) model. Deletion of ATF3 exacerbated liver damage, as evidenced by increased levels of serum ALT, intrahepatic macrophage/neutrophil trafficking, hepatocellular apoptosis, and the upregulation of pro-inflammatory mediators. ATF3 deficiency promoted mTOR and p70S6K phosphorylation, activated high mobility group box 1 (HMGB1) and TLR4, inhibited prolyl-hydroxylase 1 (PHD1), and increased HIF-1α activity, leading to Foxp3 downregulation and RORγt and IL-17A upregulation in IRI livers. Blocking mTOR or p70S6K in ATF3 knockout (KO) mice or bone marrow-derived macrophages (BMMs) downregulated HMGB1, TLR4, and HIF-1α and upregulated PHD1, increasing Foxp3 and decreasing IL-17A levels in vitro. Silencing of HIF-1α in ATF3 KO mice ameliorated IRI-induced liver damage in parallel with the downregulation of IL-17A in ATF3-deficient mice. These findings demonstrated that ATF3 deficiency activated mTOR/p70S6K/HIF-1α signaling, which was crucial for the modulation of TLR4-driven inflammatory responses and T cell development. The present study provides potential therapeutic targets for the treatment of liver IRI followed by liver transplantation.

## Introduction

Liver ischemia and reperfusion injury (IRI) is a major problem associated with liver transplantation and resection. Liver inflammatory responses induced by IR can exacerbate liver damage. Macrophages (Kupffer cells) play a critical role in triggering TLR4-mediated innate immune responses and in liver inflammation^1,2^. IR-induced liver inflammation leads to the release of endogenous damage-associated molecular pattern (DAMP) molecules, which activate the TLR4 signaling cascade on Kupffer cells and the release of pro-inflammatory cytokines leading to the activation of T cells^3–5^. Recent studies showed that T cells can differentiate into IL-17-producing cells, a distinct CD4^+^ T cell lineage that is independent from Th1 or Th2 cell development^6,7^. Th17 cells contribute to the inflammatory response by mediating the recruitment of macrophages and neutrophils to injured tissues^7^. Moreover, RORγt-expressing (RORγt^+^) T cells are the main source of Th17-producing cells during the early phase of liver IRI^8^. We previously demonstrated that RORγt/IL-17A^+^-expressing T cells played a crucial role in mediating hepatic IRI^9^.

Activating transcription factor 3 (ATF3), a basic leucine zipper (bZIP) DNA binding protein, is a member of the ATF/cAMP responsive element binding protein (CREB) family of transcription factors. Under normal conditions, ATF3 is expressed at minimal levels. However, ATF3 can be induced by various stress signals including ischemia^10^, ER stress^11^, endotoxins, and cytokines^12^. ATF3 is rapidly and preferentially induced during the early stage of the inflammatory response in organ IRI, such as in the kidney^13,14^ and brain^15^. Overexpression of ATF3 inhibits oxidative stress-induced apoptotic cell death in renal cells^13^, whereas disruption of ATF3 increases pro-inflammatory cytokine release, leading to increased susceptibility to endotoxic shock-induced cell death^16^.

The mechanistic target of rapamycin (mTOR) forms two distinct multi-protein complexes, mTOR complex1 (mTORC1) and mTOR complex2 (mTORC2)^17^. As anatypical serine/threonine kinase, mTOR plays important roles in the regulation of metabolism, cell growth, and proliferation^18^. Constitutive mTORC1 activation in macrophages promotes M1 and impairs alternative M2 polarization to enhance the inflammatory response in vitro and in vivo^19,20^. Inhibition of mTORC1 reduces LPS-induced pro-inflammatory cytokine production by suppressing NF-κB activation in macrophages^21^. Moreover, increased mTOR activity promotes T helper (Th) cell responses by reprograming metabolic processes^22^. Loss of mTOR results in failure of effector CD4^+^ T cell differentiation, whereas it induces forkhead box protein 3 (Foxp3)^+^ regulatory T cells (Tregs)^23^. Studies show that mTOR is a key regulatory factor for Th17 differentiation^24^.

Hypoxia-inducible factors (HIFs) are transcription factors that respond to low oxygen concentration or hypoxia. HIF-1 is a basic helix-loop-helix-PAS heterodimer composed of an alpha and a beta subunit^25^. HIF-1 alpha subunit (HIF-1α) is regulated by prolyl-hydroxylase domain (PHD) proteins. Under normoxia, PHD enzymes catalyze the hydroxylation of two highly conserved proline residues within the oxygen-dependent degradation (ODD) domain of HIF-1α by the E3 ubiquitin ligase von Hippel–Lindau (VHL)-mediated ubiquitin–proteasome pathway^26^. However, reduced PHD activity results in rapid HIF-α accumulation, nuclear translocation, and activation of hypoxia targeting genes under hypoxic conditions^26^. HIF-1α has a pivotal regulatory function in innate and adaptive immune cells. Disruption of myeloid-specific HIF-1α inhibits inflammatory responses by impairing macrophage aggregation and invasion^27^. HIF-1α deletion in T cells also reduces inflammatory responses by promoting Foxp3^+^ Treg and inhibiting TH17 cell differentiation^28^. Moreover, the absence of the mTOR signaling motif diminishes HIF-1α activity during hypoxia^29^, implying a mechanistic link between mTOR signaling and HIF-1α activity during hypoxia. Despite the known role of ATF3 in controlling innate inflammatory responses and the involvement of HIF-1α in mTOR signaling, the exact mechanisms by which ATF3 regulates innate immunity and adaptive T cell development in IR-triggered liver inflammation remain largely unknown.

In the present study, we showed that ATF3 deficiency aggravated IR-induced liver inflammation by activating of mTOR/p70S6K signaling and increasing TLR4-driven inflammatory responses. Activation of mTOR upregulated HIF-1α, whereas inhibiting PHD1 activity reduced Foxp3^+^ Tregs and promoted Th17 cell differentiation in IR-induced liver injury. These data indicated that ATF3-mediated mTOR/p70S6K//HIF-1α signaling is a novel regulator of innate and adaptive immunity in IR-induced liver injury.

## Results

### ATF3 deficiency exacerbates hepatocellular damage in IR-induced liver injury

**Fig. 1:**
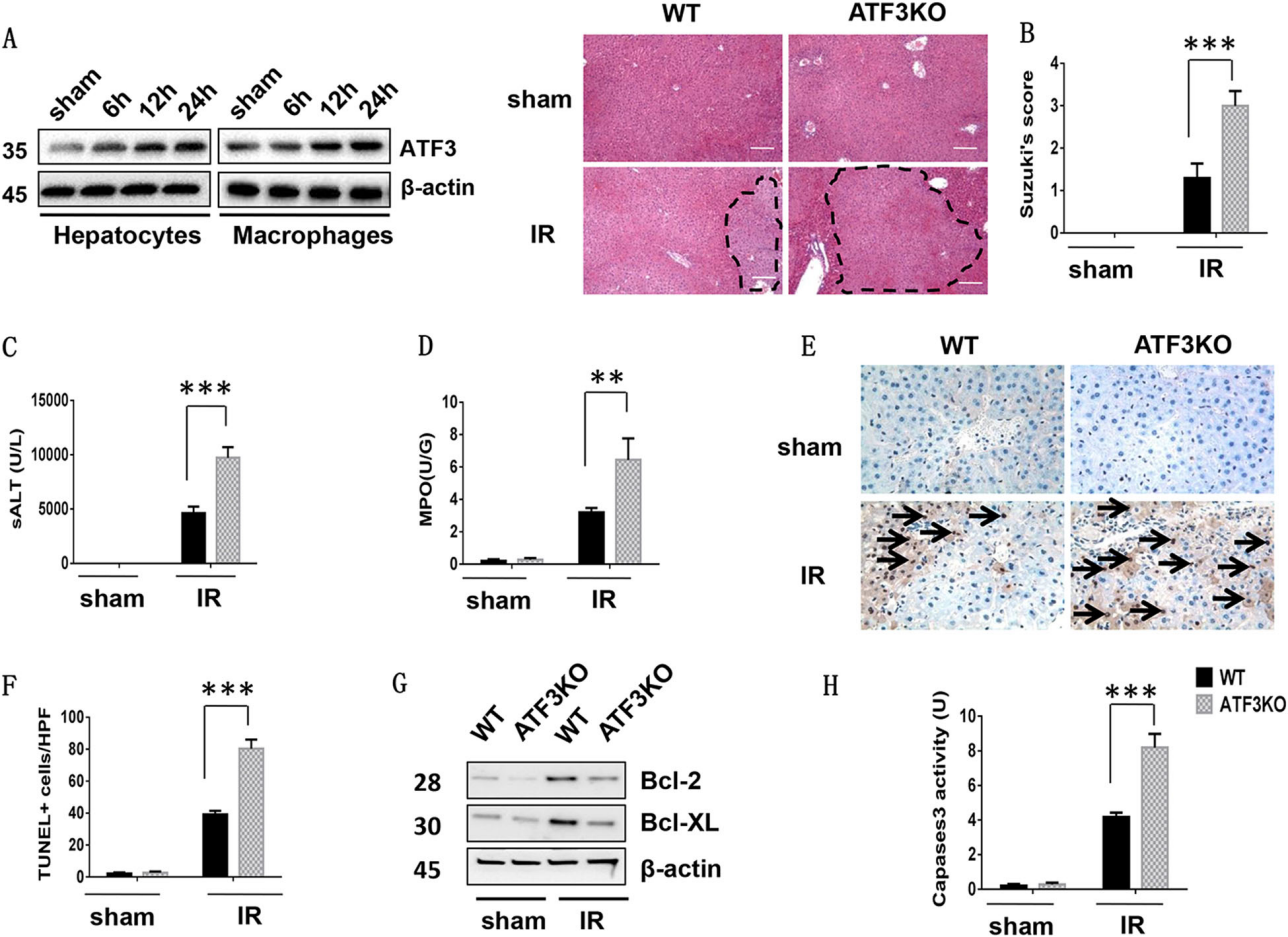
ATF3 deficiency exacerbates hepatocellular damage in IR-induced liver injury. Mice were subjected to 90 min of partial liver warm ischemia, followed by 6 h of reperfusion. **a** Western blot analysis of ATF3 protein expression in hepatocytes and macrophages during IR. Representative histological staining (H&E) of ischemic liver tissue (*n* = 4–6/group). Original magnification x100. Scale bars = 50 μm. **b** Liver damage, evaluated by Suzuki’s score. ***p < 0.001. **c** Hepatocellular function, as assessed by serum ALT levels (IU/L). Results are expressed as the mean ± SD (*n* = 4–6 samples/group), ***p < 0.001. **d** Liver neutrophil accumulation, as determined by MPO activity (U/g). Mean ± SD (representative of 4–6 mice/group). **p < 0.01. **e**, **f** Liver apoptosis analyzed by TUNEL staining. Results were scored semi-quantitatively by averaging the number of apoptotic cells (mean ± SD) per field at ×400 magnification. Representative of 4–6 mice/group, ***p < 0.001. **g** Western blot analysis of BCL-2 and BCL-xL. β-actin served as an internal control. Data are representative of three experiments. **h** Caspase-3 activity. Results are expressed as the mean ± SD (*n* = 4–6 samples/group), ***p < 0.001

To determine the effects of ATF3 in different cells on liver IRI, the expression of ATF3 was detected in hepatocytes and infiltrating macrophages at various time points after reperfusion (Fig. 1a and Supplemental Figure 1). Then, hepatocellular function was assessed in mouse livers subjected to 90 min of warm ischemia followed by 6 h of reperfusion^30^. The livers of ATF3 KO mice showed severe edema, sinusoidal congestion, and necrosis (Fig. 1a, b, score = 2.98 ± 0.35). In contrast, the livers of WT mice showed mild to moderate edema and sinusoidal congestion (Fig. 1a, b, score = 1.3 ± 0.34, *p* < 0.001). The levels of serum ALT (IU/L) were significantly higher in ATF3 KO mice than in the WT controls (Fig. 1c, 9736 ± 973 vs. 4634 ± 603, *p* < 0.001). The results of MPO assay, showed that hepatic neutrophil activity (U/g), was 3.2 ± 0.27 in the WT and 6.45 ± 1.32 in the ATF3 KO group (Fig. 1d, *p* = 0.004). Consistent with these data, ATF3 KO increased the frequency of TUNEL^+^ cells in ischemic livers compared with that in the WT controls (Fig. 1e, f, 80.4 ± 5.68 vs. 39.2 ± 2.28; *p* < 0.001). Unlike the WT controls, the protein expression of anti-apoptotic proteins (Bcl-2 and BCL-xL) was decreased in ATF3 KO livers (Fig. 1g). This was confirmed by increased caspase-3 activity in ATF3 KO but not in WT controls (Fig. 1h). These results indicated that knockdown of ATF3 exacerbated IR-induced liver damage.

### ATF3 deficiency increases macrophage/neutrophil trafficking, promotes mTOR and TLR4 activation, and induces HIF-1α signaling and T cell differentiation in IR-induced liver injury

**Fig. 2:**
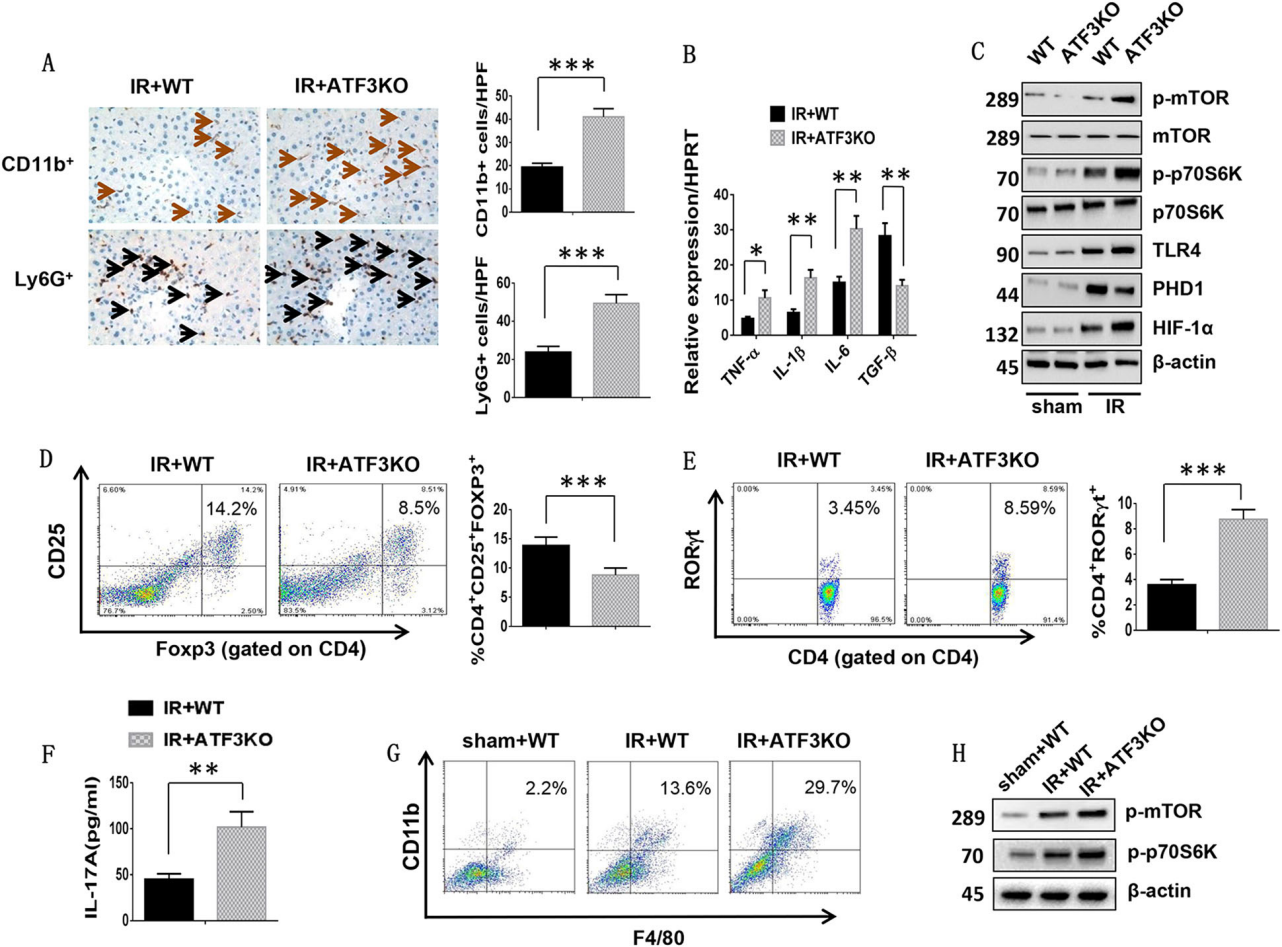
ATF3 deficiency increases macrophage/neutrophil trafficking, promotes mTOR and TLR4 activation, induces HIF-1α signaling and T cell differentiation in IR-induced liver injury. **a** Liver CD11b^+^ macrophages and Ly6G^+^ neutrophils were detected by immunohistochemistry. Results were scored semi-quantitatively by averaging the number of positively stained cells (mean ± SD)/field at ×400 magnification. Representative of 4–6 mice/group. ***p < 0.001. **b** Quantitative RT-PCR-assisted detection of TNF-α, IL-1β, IL-6, and TGF-β expression. Mean ± SD (*n* = 3-4 samples/group). *p < 0.05, **p < 0.01. **c** Western blot analysis of phosphorylated mTOR, phosphorylated p70S6K, PHD1, HIF-1α, and TLR4. β-actin served as an internal control. Data are representative of three experiments. **d**, **e** Foxp3 and RORγt expression in spleen T cells was evaluated by flow cytometry. Representative of three separate experiments. ***p < 0.001. **f** ELISA analysis of serum IL-17A levels. Mean ± SD (*n* = 3–4 samples/group). **p < 0.01. **g** Cells were stained with fluorochrome-conjugated anti-F4/80 or -CD11b. F4/80 and CD11b double-positive cells were identified as infiltrating macrophages. **h** Western blot analysis of phosphorylated mTOR and phosphorylated p70S6K in infiltrating macrophages. β-actin served as an internal control. Data are representative of three experiments

To determine whether ATF3 affected inflammatory cell recruitment in ischemic livers, CD11b^+^ macrophages and Ly6G^+^ neutrophils were detected by immunohistochemistry. CD11b^+^ macrophages and Ly6G^+^ neutrophils were increased in ATF3 KO but not in WT mice (Fig. 2a, 41 ± 3.53 vs. 19.4 ± 1.67, *p* < 0.001; 49.4 ± 4.56 vs. 23.8 ± 3.03, *p* < 0.001, respectively). ATF3 KO upregulated TNF-α, IL-1β, and IL-6 and downregulated TGF-β expression in ischemic livers compared with the WT controls (Fig. 2b). The protein expression of phospho-mTOR, phospho-p70S6K, and TLR4 was upregulated in parallel with PHD1 downregulation and HIF-α upregulation in ATF3 KO livers compared with WT livers (Fig. 2c). In addition, ATF3 KO significantly reduced the percentage of splenic CD4^+^CD25^+^Foxp3^+^ Tregs (Fig. 2d, 8.8 ± 1.18 vs. 13.86 ± 1.42, *p* < 0.001) and increased CD4^+^RoRγt^+^ TH17 cells (Fig. 2e, 8.75 ± 0.77 vs. 3.59 ± 0.41, *p* < 0.001), and this was accompanied by increased serum levels of IL-17A (Fig. 2f, 101.75 ± 16.8 vs. 45 ± 6.05, *p* = 0.003) compared with the WT controls. Finally, F4/80 and CD11b double-positive macrophages were isolated from normal (sham) and IR livers. Western blot analysis showed that the protein expression of phospho-mTOR and phospho-p70S6K in macrophages was higher in ATF3 KO than in WT livers (Fig. 2g, h). These results suggested that ATF3 played an important role in the regulation of innate TLR4 and adaptive T cell differentiation during liver inflammatory injury.

### Inhibition of mTOR signaling ameliorates ATF3 deficiency-mediated liver damage in IR-induced liver injury

**Fig. 3:**
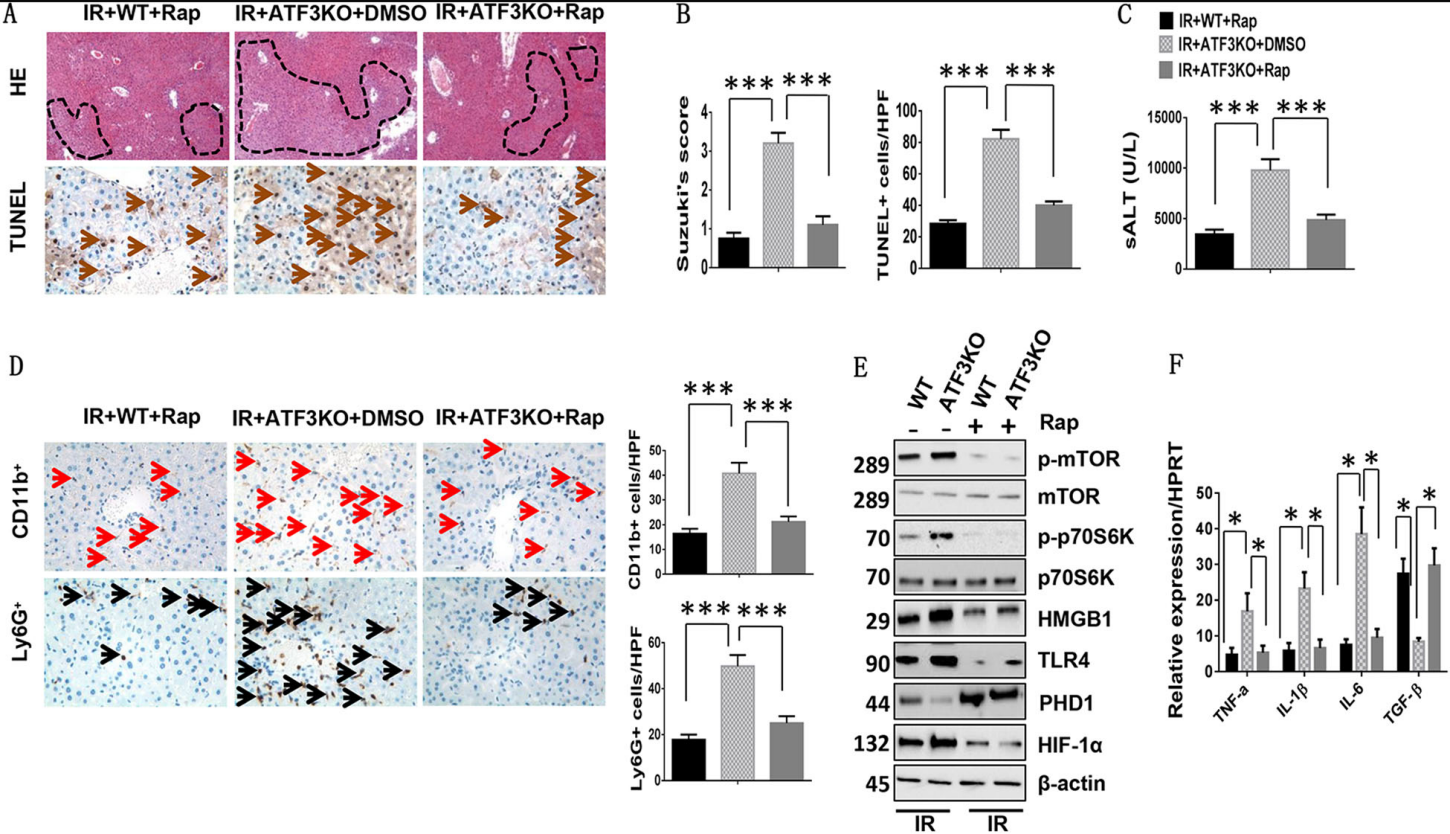
Inhibition of mTOR signaling ameliorates ATF3 deficiency-mediated liver damage in IR-induced liver injury. Mice were injected with the mTOR inhibitor rapamycin (Rap) or DMSO vehicle at 60 min prior to ischemia. **a** Representative histological staining (H&E, original magnification ×100) and TUNEL staining of ischemic liver tissue (4–6 mice/group). **b** Liver damage, as evaluated by Suzuki’s score. ***p < 0.001. TUNEL staining, results were scored semi-quantitatively by averaging the number of apoptotic cells (mean ± SD) per field at ×400 magnification. ***p < 0.001. **c** Hepatocellular function, as assessed by serum ALT levels (IU/L). Results are expressed as the mean ± SD (*n* = 4–6 samples/group). ***p < 0.001. **d** Liver CD11b^+^ macrophages and Ly6G^+^ neutrophils were detected by immunohistochemistry. Results were scored semi-quantitatively by averaging the number of positively stained cells (mean ± SD)/field at ×400 magnification. Representative of 4–6 mice/group. ***p < 0.001. **e** Western blot analysis of phosphorylated mTOR, phosphorylated p70S6K, PHD1, HIF-1α, HMGB1, and TLR4. β-actin served as an internal control. Data are representative of three experiments. **f** Quantitative RT-PCR-assisted detection of TNF-α, IL-1β, IL-6, and TGF-β expression. Mean ± SD (*n* = 3–4 samples/group). *p < 0.05

To test whether mTOR played a role in ATF3-mediated immune regulation during liver IRI, mTOR activity in ischemic livers was inhibitd by rapamycin. Pretreatment of ATF3 KO mice with rapamycin significantly improved edema, sinusoidal congestion/cytoplasmic vacuolization, and necrosis (Fig. 3a, b, 1.1 ± 0.22 vs. 3.2 ± 0.27, *p* < 0.001), and decreased the frequency of TUNEL^+^ cells (Fig. 3a, b, 40 ± 2.54 vs. 82.2 ± 5.8, *p* < 0.001) in ischemic livers compared with the DMSO vehicle controls. Consistent with the histological data, serum ALT levels (IU/L) were significantly lower in rapamycin-treated ATF3 KO mice than in the DMSO controls (Fig. 3c, 4852 ± 536 vs. 9786 ± 1092, *p* < 0.001). Moreover, rapamycin treatment in ATF3 KO mice reduced liver CD11b^+^ macrophage (Fig. 3d, 21.2 ± 2.16 vs. 40.8 ± 4.32, *p* < 0.001) and Ly6G^+^ neutrophil recruitment (Fig. 3d, 24.5 ± 0.29 vs. 49.8 ± 4.81, *p* < 0.001) compared with the DMSO-treated controls. The protein expression of phospho-mTOR, phospho-p70S6K, HMGB1, and TLR4 was decreased, whereas PHD1 was upregulated and HIF-1α was downregulated (Fig. 3e), and this was accompanied by the downregulation of TNF-α, IL-1β, and IL-6 and the upregulation of TGF-β expression in rapamycin-treated livers (Fig. 3f) compared with the DMSO-treated controls. These findings suggested that mTOR was critical for ATF3-mediated immune regulation during liver IRI.

### Blocking ATF3 deficiency-induced mTOR signaling inhibits TLR4 and HIF-1α signaling, promotes Foxp3^+^ Tregs, and inhibits Th17 cell differentiation in vitro

**Fig. 4:**
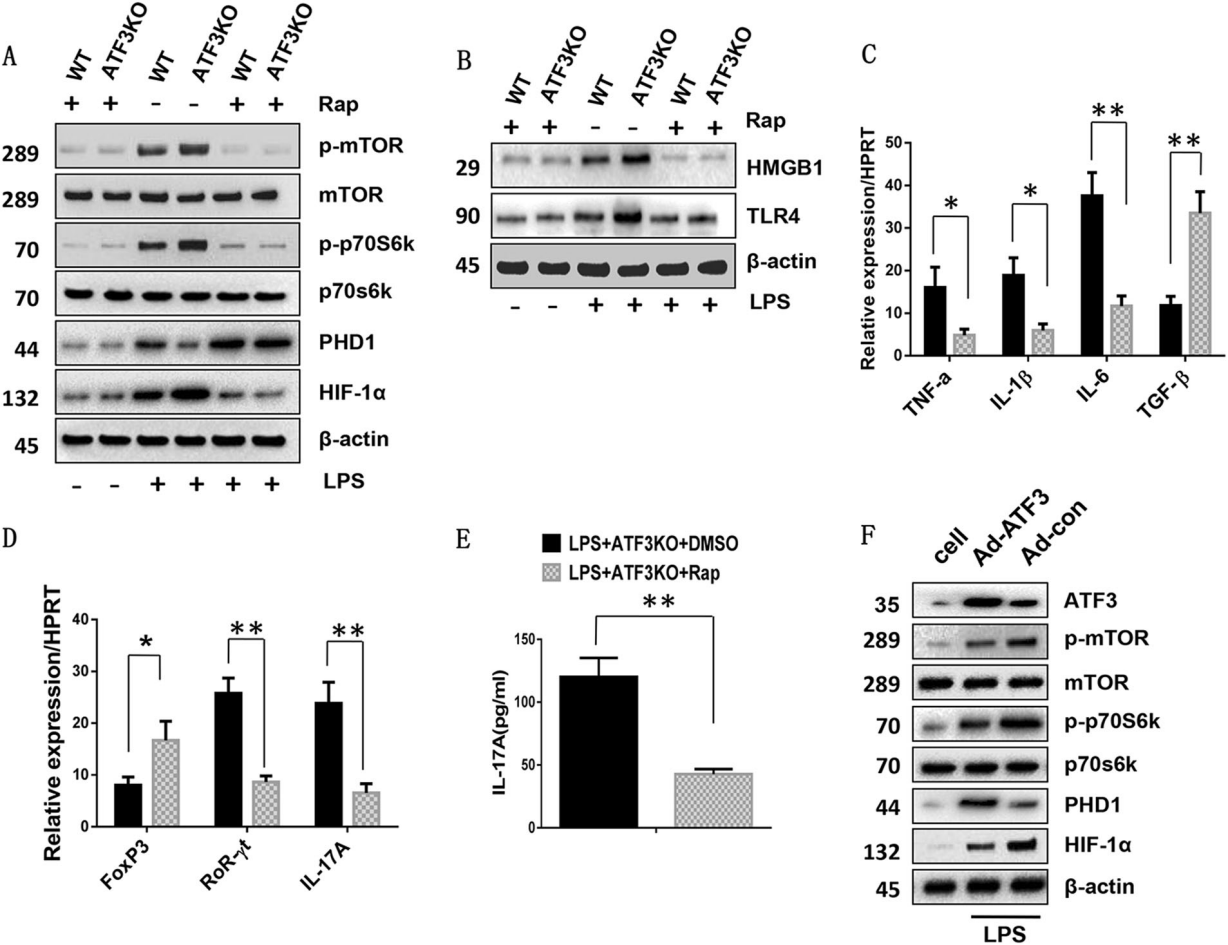
Blocking ATF3 deficiency-induced mTOR signaling inhibits TLR4 and HIF-1α signaling, promotes Foxp3^+^ Tregs and inhibits Th17 cell differentiation in vitro. Bone marrow-derived macrophages (BMMs) were isolated from WT and ATF3 KO mice and pretreated with Rap or DMSO vehicle controls, and then co-cultured with splenic CD4^+^ T cells after LPS stimulation for 6 h. **a**, **b** Western blot analysis of phosphorylated mTOR, phosphorylated p70S6K, PHD1, HIF-1α, HMGB1, and TLR4 in LPS-stimulated BMMs. β-actin served as an internal control. **c** Quantitative RT-PCR-assisted detection of TNF-α, IL-1β, IL-6, and TGF-β in LPS-stimulated BMMs. Mean ± SD (*n* = 3–4 samples/group). *p < 0.05, **p < 0.01. **d** Quantitative RT-PCR-assisted detection of Foxp3, RORγt, and IL-17A in splenic CD4^+^ T cells after co-culture with Rap or DMSO-pretreated BMMs. Mean ± SD (*n* = 3–4 samples/group). *p < 0.05, **p < 0.01. **e** ELISA analysis of IL-17A levels in co-culture supernatants. Mean ± SD (*n* = 3–4 samples/group). **p < 0.01. **f** Western blot analysis of ATF3, phosphorylated mTOR, phosphorylated p70S6K, PHD1, and HIF-1α in LPS-stimulated BMMs followed by Ad-ATF3 or Ad-con-pretreatment. β-actin served as an internal control. Data are representative of three experiments

To determine the mechanisms underlying the role of ATF3 deficiency-induced mTOR signaling in the regulation of innate TLR4, HIF-1α signaling and adaptive T cells differentiation, mTOR activity was inhibited by rapamycin in BMMs from ATF3 KO mice. ATF3 KO upregulated phospho-mTOR and phospho-p70S6K, downregulated PHD1 and upregulated HIF-1α in LPS-stimulated BMMs after DMSO treatment. However, inhibition of mTOR by rapamycin pretreatment downregulated phospho-mTOR, phospho-p70S6K, and HIF-1α and upregulated PHD1 after LPS stimulation (Fig. 4a). Moreover, rapamycin treatment downregulated HMGB1 and TLR4 in ATF3-deficient BMMs compared with the DMSO-treated controls (Fig. 4b). Decreased TNF-α, IL-1β, and IL-6 levels and increased TGF-β mRNA levels were observed in rapamycin-treated cells but not in DMSO-treated controls after LPS stimulation (Fig. 4c). To determine whether macrophage ATF3 deficiency affected mTOR/HIF-1α signaling and T cell differentiation, rapamycin-pretreated ATF3-deficient BMMs were co-cultured with spleen CD4^+^ T cells after LPS stimulation. Inhibition of mTOR in ATF3-deficient BMMs decreased the mRNA levels of RORγt and IL-17A and increased Foxp3 levels in spleen CD4^+^ T cells (Fig. 4d). Consistent with these data, ELISA-assessed IL-17A levels were significantly decreased in rapamycin-pretreated groups compared with those in DMSO-treated controls in co-culture supernatants (Fig. 4e). To further determine whether PHD1, HIF-1a, S6K, and mTOR were specifically modulated by ATF3, we used Ad-ATF3 or Ad-con to overexpress ATF3 in BMMs from WT mice. ATF3 overexpression inhibited the expression of phospho-mTOR, phospho-p70S6K, upregulated PHD1, and downregulated HIF-1α in LPS-stimulated BMMs after Ad-ATF3 or Ad-con treatment (Fig. 4f). These results suggested that macrophage ATF3 regulated innate TLR4 and adaptive T cell differentiation via the mTOR/HIF-1α-mediated signaling pathway.

### p70S6K mediates mTOR signaling in ATF3-mediated immune regulation in vitro

**Fig. 5:**
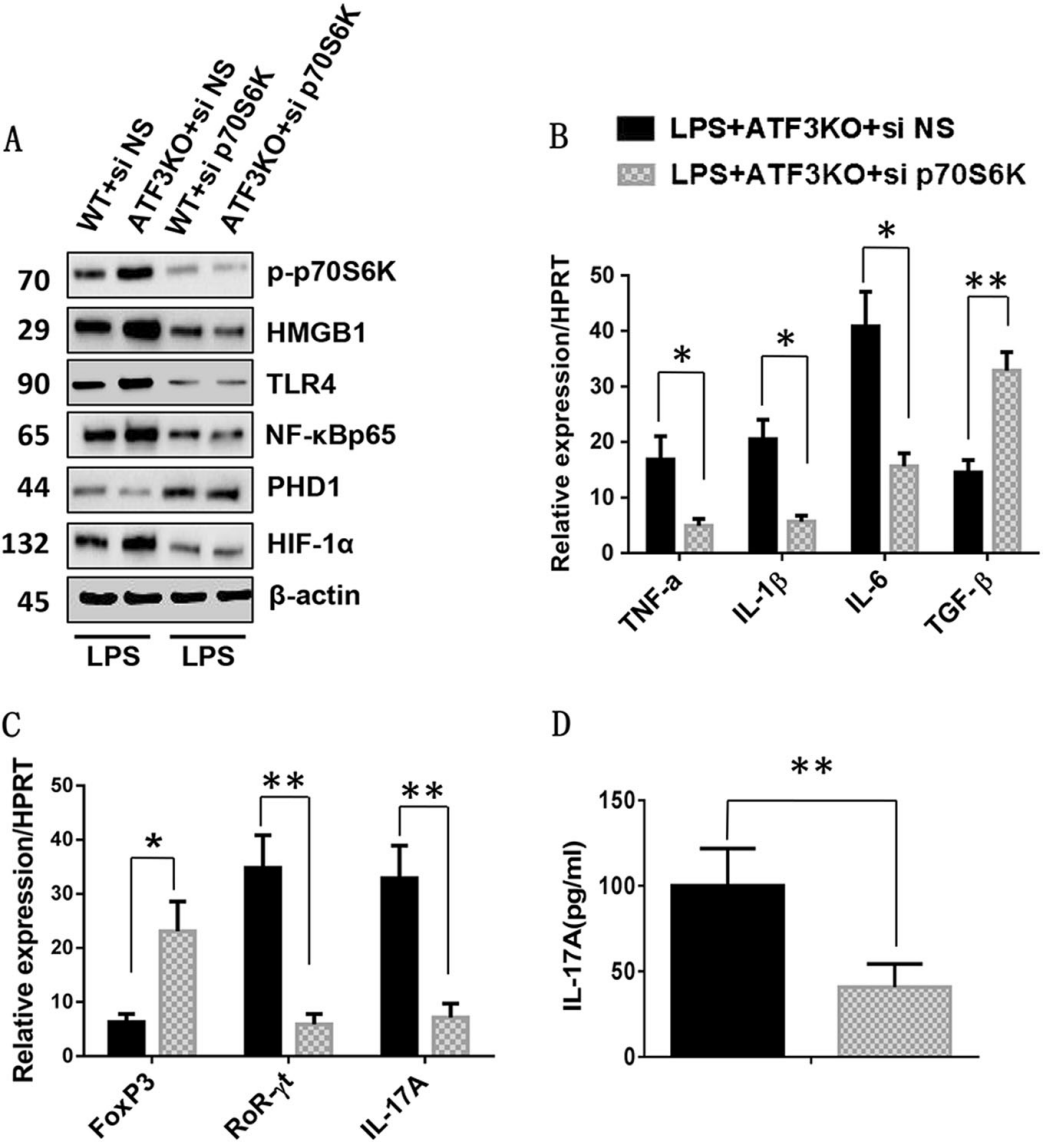
p70S6K mediates mTOR signaling in ATF3-mediated immune regulation in vitro. Bone marrow-derived macrophages (BMMs) were isolated from WT and ATF3 KO mice and transfected with p70S6K siRNA (si-p70S6K), and then co-cultured with splenic CD4^+^ T cells after LPS stimulation for 6 h. Non-specific (NS) siRNA served as a control. **a** Western blot analysis of phosphorylated p70S6K, PHD1, HIF-1α, HMGB1, TLR4, and NF-κB in LPS-stimulated BMMs. β-actin served as an internal control. **b** Quantitative RT-PCR detection of TNF-α, IL-1β, IL-6, and TGF-β in LPS-stimulated BMMs. Mean ± SD (*n* = 3–4 samples/group). *p < 0.05, **p < 0.01. **c** Quantitative RT-PCR detection of Foxp3, RORγt, and IL-17A in splenic CD4^+^ T cells after co-culture with p70S6K siRNA or NS siRNA-pretreated BMMs. Mean ± SD (*n* = 3–4 samples/group). *p < 0.05, **p < 0.01. **d** ELISA analysis of IL-17A levels in co-culture supernatants. Mean ± SD (*n* = 3–4 samples/group). **p < 0.01

As the phosphorylation of S6K acted downstream of mTOR activation and played an important role in cell metabolism and transcriptional regulation^31^, we next investigated the functional role of macrophage p70S6K in ATF3-mediated immune regulation in cell cultures. Unlike the non-specific (NS) siRNA-treated controls, siRNA-mediated knockdown of p70S6K (si-p70S6K) in ATF3-deficient BMMs downregulated HMGB1, TLR4, and NF-κB, upregulated PHD1, and downregulated HIF-1α after LPS stimulation (Fig. 5a). Treatment of ATF3-deficient BMMs with si-p70S6K decreased the mRNA levels of TNF-α, IL-1β, and IL-6 and increased those of TGF-β in response to LPS stimulation compared with the NS siRNA-treated controls (Fig. 5b). Using macrophage (BMM)/spleen CD4^+^ T cell co-culture system, p70S6K siRNA treatment in BMMs upregulated Foxp3 and downregulated RORγt and IL-17A expression in spleen CD4^+^ T cells (Fig. 5c), and this was accompanied by reduced IL-17A levels after co-culture (Fig. 5d). These data indicated that mTOR contributed to ATF3-mediated immune regulation in a p70S6K-dependent manner.

### Disruption of HIF-1α ameliorates ATF3 deficiency-mediated liver damage and inhibits Th17 cell differentiation in vivo

**Fig. 6:**
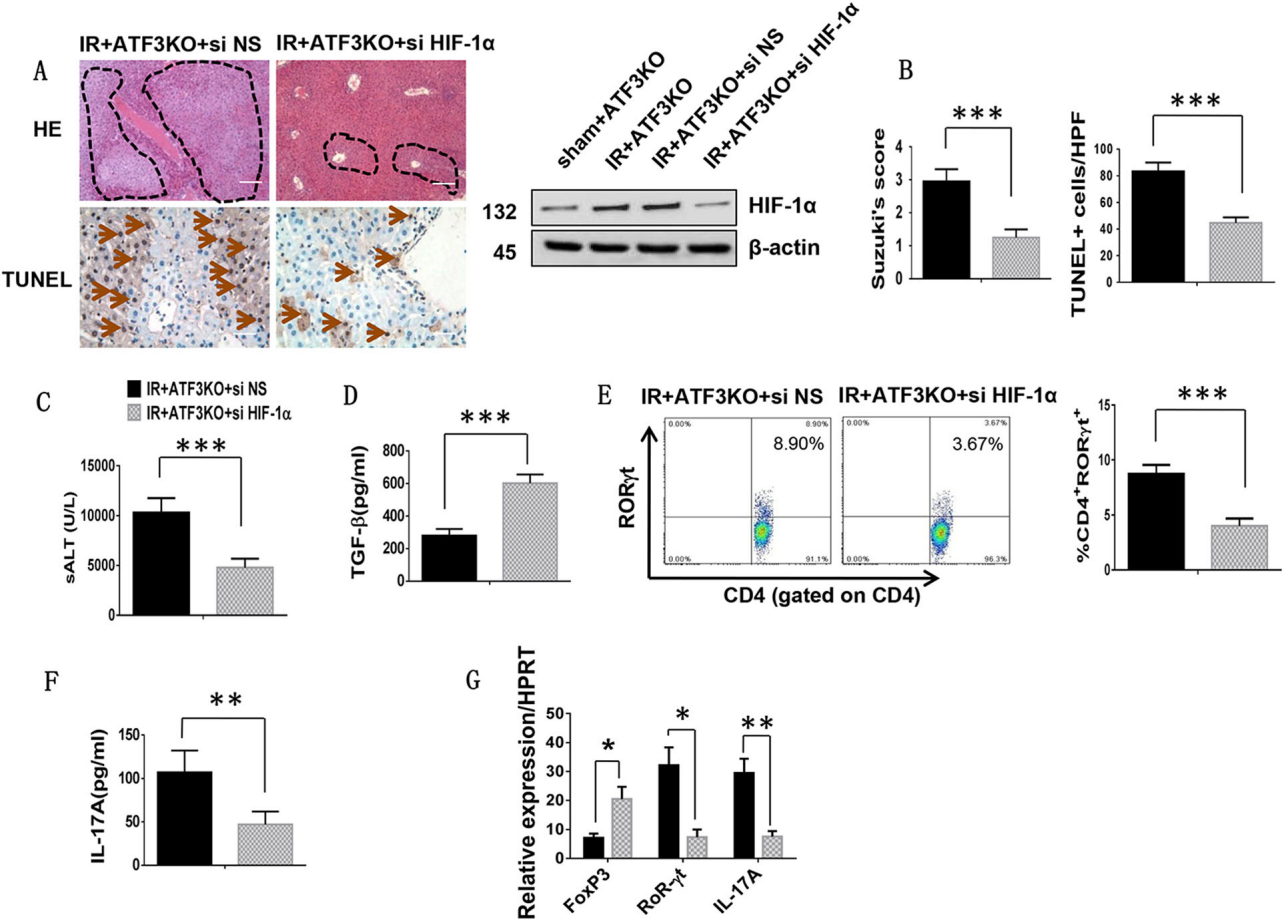
Disruption of HIF-1α ameliorates ATF3 deficiency-mediated liver damage and inhibits Th17 cell differentiation in vivo. ATF3 KO mice were injected via the tail vein with a mannose-mediated HIF-1α siRNA or NS siRNA at 4 h prior to ischemia. **a** Representative histological staining (H&E, original magnification ×100) and TUNEL staining of ischemic liver tissue (4–6 mice/group). Scale bars = 50 μm. Western blot analysis of HIF-1α in HIF-1α siRNA or NS siRNA-pretreated livers subjected to IR. β-actin served as an internal control. **b** Liver damage, as evaluated by Suzuki’s score. ***p < 0.001. TUNEL staining, results were scored semi-quantitatively by averaging the number of apoptotic cells (mean ± SD) per field at ×400 magnification. ***p < 0.001. **c** Hepatocellular function, as assessed by serum ALT levels (IU/L). Results are expressed as the mean ± SD (*n* = 4–6 samples/group). ***p < 0.001. **d** ELISA analysis of serum TGF-β levels. Mean ± SD (*n* = 3–4 samples/group). ***p < 0.001. **e** RORγt expression in spleen T cells was evaluated by flow cytometry. Representative of three separate experiments. ***p < 0.001. **f** ELISA analysis of serum IL-17A levels. Mean ± SD (*n* = 3–4 samples/group). **p < 0.01. **g** Foxp3, RORγt, and IL-17A in mouse livers. Mean ± SD (*n* = 3–4 samples/group). *p < 0.05, **p < 0.01

**Fig. 7:**
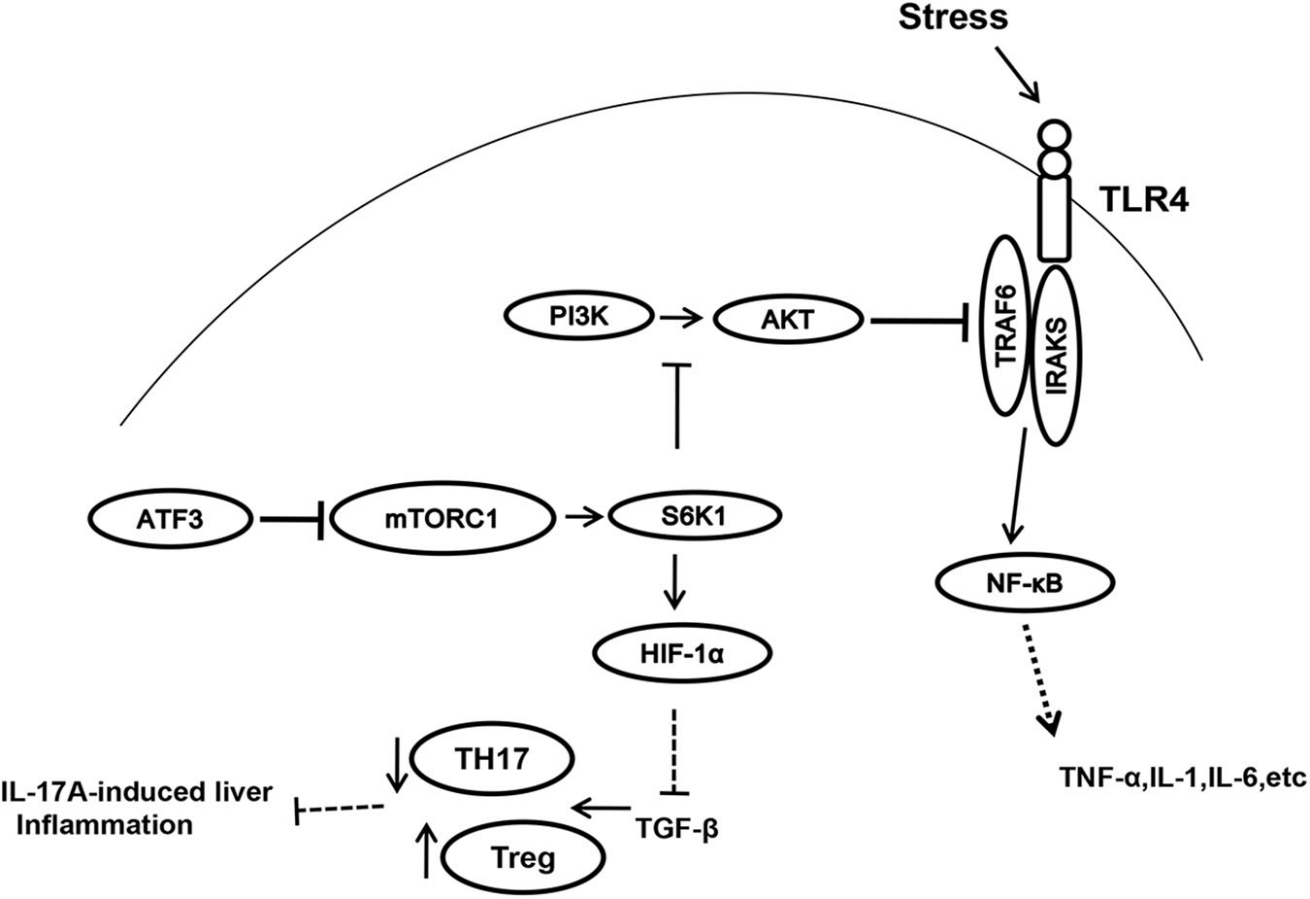
Schematic illustration of the molecular mechanisms underlying the role of mTOR/p70S6K/HIF-1α signaling pathway in the regulation of TLR4 inflammatory signaling and TH17 cells in liver IRI

Having demonstrated the important role of macrophage p70S6K/HIF-1α signaling in the modulation of innate and adaptive immunity in vitro, we next investigated whether disruption of macrophage HIF-1α in ATF3 KO mice affected inflammatory responses and T cell differentiation in mouse liver IRI. An HIF-1α siRNA with an in vivo mannose-mediated delivery system, which enhances delivery to cells expressing a mannose-specific membrane receptor, was used to transfect to macrophages^32^. First, we assessed the extent of HIF-1a downregulation after HIF-1α siRNA or NS siRNA treatment, which showed that pretreatment with HIF-1α siRNA markedly decreased the protein levels of HIF-1α (Fig. 6a). The livers of ATF3 KO mice treated with NS siRNA displayed significant edema, severe sinusoidal congestion/cytoplasmic vacuolization, and extensive necrosis (Fig. 6a, b, score = 2.95 ± 0.37). However, the livers of ATF3 KO mice treated with mannose-mediated HIF-1α siRNA showed mild to moderate edema without necrosis (Fig. 6a, b, score = 1.25 ± 0.25, *p* < 0.001), and a lower frequency of TUNEL^+^ cells than the NS siRNA-treated controls (Fig. 6a, 83.4 ± 6.54 vs. 44.6 ± 4.2, *p* < 0.001). These data were consistent with the results of hepatocellular function analysis, which showed that mannose-mediated HIF-1α siRNA treatment in ATF3 KO mice decreased sALT levels compared with those in the NS siRNA-treated controls (Fig. 6c, 10,304 ± 1449 vs. 4798 ± 883, *p* < 0.001). Moreover, HIF-1α siRNA treatment in ATF3 KO livers increased serum TGF-β release (Fig. 6d, 281.2 ± 39.55 vs. 602.6 ± 53.04, *p* < 0.001), and this was accompanied by a reduction in the percentage of splenic CD4^+^RoRγt^+^ TH17 cells (Fig. 6e, 8.74 ± 0.82 vs. 4.01 ± 0.67, *p* < 0.001) and serum IL-17A levels (Fig. 6f, 107 ± 25.2 vs. 47 ± 14.9, *p* = 0.009) compared with the NS siRNA-treated controls. RORγt and IL-17A mRNA levels were reduced, whereas Foxp3 levels were increased in HIF-1α siRNA-treated groups but not the NS siRNA-treated controls (Fig. 6g). These results suggested that macrophage HIF-1α signaling was essential for modulating Th17 cell differentiation and inflammatory responses in ATF3-mediated immune regulation (Fig. 7).

## Discussion

The present study is the first to demonstrate that ATF3-mediated mTOR/p70S6K/HIF-1α signaling is crucial for orchestrating inflammatory responses in IR-induced liver injury. The data can be summarized as follows: (i) ATF3 deficiency exacerbated IR-induced liver damage, increased macrophage/neutrophil trafficking, promoted mTOR and its downstream p70S6K, and activated TLR4/NF-κB; and (ii) ATF3-mediated mTOR/p70S6K induced HIF-1α signaling, which was essential for T cell differentiation in liver IRI. These results highlighted the role of ATF3-mediated mTOR/p70S6K/HIF-1α signaling in the regulation of innate and adaptive immunity during liver inflammatory injury.

ATF3, an oxidative stress-responsive transcription factor, is associated with a variety of immune and inflammatory diseases^13,33^. Although previous studies showed that ATF3 modulates inflammatory responses by inhibiting TLR4-mediated inflammatory cytokines^34,35^, little is known about the exact mechanism by which ATF3 regulates innate TLR4 and adaptive T cell differentiation in IR-induced liver injury. In addition, ATF3 has multiple roles in neuroprotection and neuroregeneration^36,37^. In the current study, ATF3 deletion increased the inflammatory response, as evidenced by the exacerbation of IR-induced liver damage and increased hepatocellular apoptosis and intrahepatic macrophage/neutrophil accumulation. Moreover, ATF3 deficiency activated mTOR, p70S6K, TLR4, and HIF-1α, and downregulated PHD1. Disruption of ATF3 downregulated Foxp3 and upregulated RORγt-mediated IL-17A expression in IR-induced liver inflammation. These results underscored the importance of ATF3 as a negative transcriptional regulator of innate TLR4 and T cell differentiation during liver inflammatory injury.

The molecular mechanisms underlying the role of ATF3 in regulating innate TLR4 and adaptive T cell differentiation may involve in multiple cellular and molecular signaling pathways. mTOR, serine/threonine kinase, function as a core component of two distinct protein complexes, mTORC1 and mTORC2, which regulate different cellular functions^38^, including cell growth, lipogenesis, protein synthesis, and transcription^39,40^. Constitutive mTORC1 activation promotes M1 macrophage polarization and increases inflammatory responses after LPS stimulation^19,20^. Activation of mTORC1 upregulates TLR4 signaling and pro-inflammatory cytokines in acute lung injury^41^. Moreover, mTOR is essential for T cell proliferation and differentiation^42–46^. The close association of ATF3 with mTOR was reported previously^47^. Consistent with these results, we found that ATF3 knockout promoted mTOR activity, whereas inhibition of mTOR ameliorated IR-induced liver injury and reduced HMGB1 and TLR4 activation, suggesting that mTOR signaling mediated the role of ATF3 in the regulation of innate TLR4 during liver IRI.

Further evidence of mTOR signaling-mediated modulation of innate TLR4 was obtained from our in vitro study. The results showed that ATF3 deficiency promoted mTOR and its downstream target gene p70S6K phosphorylation in LPS-stimulated BMMs. However, inhibition of mTOR in ATF3-deficient BMMs reduced p70S6K activity and downregulated HMGB1 and TLR4, and this was accompanied by the downregulation of pro-inflammatory cytokines. Indeed, p70S6K functions as part of the mTOR signaling pathway. Phosphorylation of p70S6K is dependent on mTOR, specifically on mTORC1. The mTORC1-S6K axis controls a variety of cellular processes and contributes to cell development and disease^31^. Disruption of TSC-mediated mTOR/S6K signaling is associated with several human genetic disorders^48^. Consistent with these findings, we found that disruption of p70S6K in ATF3-deficient BMMs inhibited TLR4-driven inflammatory responses after LPS stimulation. Notably, ATF3 deletion in LPS-stimulated BMMs reduced PHD1 activity and increased HIF-1α induction, whereas p70S6K knockdown upregulated PHD1 and downregulated HIF-1α, thus decreasing pro-inflammatory mediators in ATF3-deficient cells. These results suggested that ATF3-mediated mTOR/p70S6K signaling positively regulated HIF-1α activity during the inflammatory response.

One striking finding was that ATF3 deficiency depressed Foxp3^+^ Tregs, whereas it increased ROR^+^ Th17 cells in IR-induced liver inflammation. Although previous studies showed that mTOR is a major regulator of T cell differentiation and expansion^49^, how macrophage ATF3 affects T cell differentiation remains unknown. In the present macrophage/CD4^+^ T cell co-culture system, increased HIF-1α induction in ATF3-deficient BMMs upregulated RORγt and IL-17A and downregulated Foxp3 expression in splenic CD4^+^ T cells, and this was accompanied by increased IL-17A production. However, inhibition of mTOR by rapamycin in ATF3-deficient BMMs upregulated PHD1 and downregulated HIF-1α, resulting in increased Foxp3 and diminished RORγt and IL-17A levels. Indeed, HIF-1α can regulate innate and adaptive immune cell functions. Ablation of myeloid-specific HIF-1α suppresses inflammatory responses by inhibiting macrophage infiltration and activation^27^. The contribution of HIF-1α to the inflammatory response is dependent on NF-κB activity^50^. Moreover, HIF-1α promotes Th17 cell development by activating RORγt transcription, whereas deletion of HIF-1α in T cells promotes Foxp3^+^ Tregs and decreased RORγt^+^ Th17 cells^28^. Consistent with these results, we found that disruption of HIF-1α in ATF3 KO mice alleviated IR-induced liver damage and improved hepatic function, and this occurred in parallel with reduced RORγt-mediated Th17A levels and increased Foxp3 expression. Thus, our findings revealed an essential role for HIF-1α in the control of T cell differentiation in ATF3-mediated immune regulation during liver inflammatory injury.

The effect of macrophage ATF3-mediated mTOR signaling on the ability of HIF-1α to regulate T cell differentiation remains unclear. We showed that ATF3 deficiency increased HIF-1α induction while reducing PHD1 activity. However, inhibition of mTOR in ATF3 KO mice reduced phosphorylated p70S6K and HIF-1α and increased PHD1 in ischemic livers. This suggested a possible mechanistic link between mTOR and HIF-1α in the regulation of T cell differentiation? Indeed, HIF-1α stability is primarily modulated by PHD1 in an oxygen-dependent-manner. PHD1 acts as an oxygen-sensing enzyme and promotes HIF-1α hydroxylation and proteasomal degradation in normoxia, whereas inactivated PHD1 during hypoxia leads to the stabilization of HIF-1α and its translocation into the nucleus to activate the transcription of target genes^51^. Thus, we speculate that ATF3-mediated mTOR signaling may play an important role in the regulation of the HIF-1α-PHD1 oxygen-sensing pathway. As p70S6K activation is modulated by mTOR, p70S6K may be essential for the regulation of HIF-1α induction in the mechanism of adaptive T cell development. This was supported by our further experiments. We used a co-culture system to show that knockdown of p70S6K in ATF3-deficient BMMs increased PHD1 and reduced HIF-1α activity, and this was accompanied by increased Foxp3 and decreased RORγt-mediated IL-17A levels in splenic CD4^+^ T cells. Taken together, these data indicated that ATF3-mediated mTOR/p70S6K/HIF-1α signaling was crucial for T cell differentiation in IR-triggered liver inflammation.

In conclusion, we demonstrated that ATF3 deficiency exacerbated IR-induced liver inflammation by upregulating mTOR and its downstream target gene p70S6K, which in turn activated innate TLR4 and increased HIF-1α while reducing PHD1 activity, leading to depressed Foxp3^+^ Treg and promoting RORγt^+^ Th17 cell differentiation. The present study has increased our knowledge of the molecular mechanisms underlying the role of ATF3-mediated mTOR/p70S6K//HIF-1α signaling in the modulation of innate TLR4 and adaptive T cell differentiation, thus providing potential therapeutic targets in liver IRI followed by liver transplantation.

## Materials and methods

### Animals

WT C57BL/6 mice were purchased from the Laboratory Animal Resources of Nanjing Medical University (NMU). ATF3 knockout (KO) mice in the C57BL/6 background were previously described^34^. Male, 8-week-old WT and ATF3 KO mice were used in all experiments. This study was performed in strict accordance with the recommendations in the *Guide for the Care and Use of Laboratory Animals* published by the National Institutes of Health. The animal protocol was approved by the Institutional Animal Care & Use Committee (IACUC) of Nanjing Medical University (Protocol Number NMU08-092).

### Mouse liver IRI model

A mouse model of warm hepatic ischemia followed by reperfusion was used, as described^30^. Mice were injected with heparin (100 U/kg) and an atraumatic clip was used to interrupt the arterial/portal venous blood supply to the cephalad liver lobes. After 90 min the clip was removed, and mice were killed at 6 h of reperfusion. Mice were injected with mTOR inhibitor Rapamycin (5 mg/kg, i.p. Calbiochem, Burlington, MA) or DMSO vehicle at 60 min prior to ischemia. In some experiments, animals were injected via tail vein with HIF-1α siRNAs or non-specific (control) siRNA, (2 mg/kg) (Santa Cruz Biotechnology, Shanghai, China) mixed with mannose-conjugated polymers (Polyplus transfection™, Illkirch, France) at a ratio according to the manufacturer’s instructions 4 h prior to ischemia as described^32^.

### Hepatocellular function assay

Serum alanine aminotransferase (sALT) levels, an indicator of hepatocellular injury, were measured by an automated chemical analyzer (Olympus Automated Chemistry Analyzer AU5400, Tokyo, Japan).

### Histology

Liver sections were stained with hematoxylin and eosin (H&E). The severity of IRI was graded using Suzuki’s criteria on a scale from 0 to 4^52^. In this classification, no necrosis, congestion, or centrilobular ballooning is given a score of 0, while severe congestion and ballooning degeneration and >60% lobular necrosis is given a value of 4.

### Immunohistochemistry staining

Liver macrophages and neutrophils were detected using primary rat anti-mouse CD11b^+^ mAb (Mac-1, M1/70; BD Biosciences, San Jose, CA) or Ly6G^+^ mAb (BD Biosciences, San Diego, CA). After incubation with secondary biotinylated goat anti-rat IgG (Vector, Burlingame, CA), followed by treatment with immunoperoxidase (ABC Kit, Vector), positive cells were counted blindly in ten HPF/section (×400). For immunofluorescence, frozen liver sections were labeled with primary antibodies ATF3 and CD11b (Santa Cruz Biotechnology, CA), and then incubated with secondary Cy3-conjugated AffiniPure donkey anti-goat IgG antibody (Jackson Immunoresearch, PA). The samples were pre-mounted with VECTASHIELD medium with DAPI.

### Myeloperoxidase activity assay

The presence of myeloperoxidase (MPO) was used as an index of hepatic neutrophil accumulation^2^. The change in absorbance was measured spectrophotometrically at 655 nm. One unit of MPO activity was defined as the quantity of enzyme degrading 1 μmol peroxide/min at 25 °C/g of tissue.

### TUNEL staining

Liver sections (4 mm) were stained via terminal deoxynucleotidyl transferase dUTP nick end labeling (TUNEL) using the in situ cell death detection kit (Roche-Boehringer Mannheim, Germany) according to the manufacturer’s instructions as previously described^2^.

### Caspase-3 activity assay

Caspase-3 activity was determined by an assay kit (Calbiochem, La Jolla, CA), as previously described^2^. Liver tissues were collected and resuspended in lysis buffer containing 50 mmol/L HEPES, pH 7.4, 0.1% CHAPS, 1 mmol/L DTT, 0.1 mmol/L EDTA, and 0.1% Triton X-100. Following incubation for 30 min on ice, cell lysate was centrifuged at 16,000 × *g* for 10 min at 4 ℃, and the protein concentration in the supernatants was measured using the Bradford dye method. The supernatants were incubated with 200 μM of enzyme-specific colorimetric caspase-3 substrate at 37 °C for 2 h. Caspase-3 activity was assessed by measuring the absorbance at a wavelength of 405 nm with a plate reader. To determine cellular activity, the inhibitor-treated protein extracts and the purified caspase-3 (as a standard) were used.

### ELISA

IL-17A and TGF-β levels were measured by ELISA according to the manufacturer’s standard protocols (eBioscience, San Diego, CA). Absorbance was read on a Multiscan FC plate reader and analyzed with SkanIt for Multiscan FC software (Thermo Scientific, Schwerte, Germany).

### Quantitative RT-PCR analysis

Quantitative real-time PCR was performed using the DNA Engine with Chromo 4 Detector (MJ Research, Waltham, MA). In a final reaction volume of 25 μl, the following were added: 1× SuperMix (Platinum SYBR Green qPCR Kit; Invitrogen, San Diego, CA) cDNA and 10 μM of each primer. Amplification conditions were: 50 °C (2 min), 95 °C (5 min), followed by 40 cycles of 95 °C (15 s) and 60 °C (30 s). Primer sequences used for the amplification were shown in Supplementary Table 1.

### Western blot analysis

Protein was extracted from liver tissue or cell cultures, as described^53^. Monoclonal rabbit anti-mouse ATF3 (Santa Cruz Biotechnology, Shanghai, China), phos-mTOR, mTOR, phos-p70S6K, p70S6K, HMGB1, TLR4, NF-κB, HIF-1α, PHD1, Bcl-2, Bcl-xl, and β-actin Abs (Cell Signaling Technology, San Diego, CA) were used. The relative quantities of proteins were determined by densitometer, and expressed in absorbance units (AU).

### BMM isolation and in vitro transfection

Murine bone marrow-derived macrophages (BMMs) were generated as previously described^54^. In brief, bone marrow cells were removed from the femurs and tibias of WT and ATF3 KO mice and cultured in DMEM supplemented with 10% FCS and 20% L929-conditioned medium. Cells (1 × 10^6^/well) were cultured for 7 days and then transfected with 100 nM of p70S6K siRNA (Santa Cruz Biotechnology) using lipofectamine 2000 reagent (Thermo Fish Scientific, Carlsbad, CA). The non-specific (NS) siRNA as controls. After 24–48 h, cells were supplemented with 100 ng/ml of LPS for additional 6 h. In some experiments, BMMs were pretreated with 20 nM of rapamycin (Calbiochem) or DMSO vehicle at 60 min prior to LPS stimulation.

### Spleen CD4^+^ T cell isolation

Spleen T cells were purified using the EasySep™ mouse T cell isolation kit (STEMCELL Technologies, Vancouver, BC, Canada) according to the manufacturer’s instructions. T cells were then stimulated with anti-CD3 (1 µg/ml) and anti-CD28 (2 µg/ml) (eBioscience). CD4^+^ T cells were isolated from these cells using anti-CD4 microbeads (Miltenyi Biotec, Bergisch Gladbach, Germany) according to the manufacturer’s instructions.

### Macrophage/CD4^+^ T cell co-culture

Rapamycin or p70S6K siRNA pretreated ATF3 KO macrophages were counted to 0.5 × 10^6^ cells/ml and cultured on 60 mm plates. After the cells stimulated with LPS (100 ng/ml) for 6 h, spleen CD4^+^ T cells were then added into cultures at a macrophage/T cell ratio of 1:5. The co-cultured cells were incubated for 24 h, and then macrophages and spleen CD4^+^ T cells were harvested for the real-time PCR and western blot assay.

### Flow cytometry analysis

Spleen T cells isolated from WT, ATF3 KO, and HIF-1α siRNA or NS siRNA-treated ATF3 KO mice were stained with anti-mouse CD4-PE-Cyanine5, CD25-PE, RoRγt-PE, and Foxp3-FITC mAbs (eBioscience) according to the manufacturer’s instructions. PE-labeled rat anti-mouse IgG2a isotypes were used as negative controls. Measurements were performed using a FACS Calibur flow cytometer (BD Biosciences). Data analysis was performed using Cell Quest software. Liver NPCs were isolated from sham or IR livers, as described above^55^. A total of 1 × 10^6^ cells were incubated with purified rat anti-mouse CD16/32 for 10 min and stained with rat anti-mouse F4/80-PeCy5/PE, CD11b-FITC, and isotype-matched negative control Abs (eBioscience, San Diego, CA) were added to the cell suspension. After 20 min of incubation in the dark, the cells were washed with PBS and subjected to flow cytometric analysis with FACS Calibur (BD Biosciences). For intracellular staining of CD206 and inducible NO synthase, cells were fixed in 4% formaldehyde for 20 min after the staining of F4/80 and CD11b, and washed twice with 1× permeabilization buffer (eBioscience). After incubation with CD206-APC (BioLegend, San Diego, CA) and inducible NO synthase-PE (eBioscience) in 1× permeabilization buffer for 20 min in the dark, the cells were washed with PBS and subjected to flow cytometric analysis.

### Adenovirus gene transfer

Adenoviral vector encoding the mouse ATF3 gene (Ad-ATF3) and negative control (Ad-con) was constructed, packaged, purified, and titrated at Genechem Co. Ltd. For adenovirus-mediated gene transfer, Ad-ATF3 or Ad-con was transfected into macrophages from WT mice at a final concentration of 0.5 × 10^6^ cells/ml for 48 h. After 48 h, the overexpression efficiency of Ad-ATF3 was evaluated by western blot.

### Statistical analysis

Data are expressed as mean ± SD and analyzed by Student’s *t* tests. Per comparison, two-sided *p* values less than 0.05 were considered statistically significant. Multiple group comparisons were performed using one-way ANOVA with a post hoc test. All statistical analysis was performed using SPSS-3 software.

## Electronic supplementary material

Supplementary Figure 1

Supplementary Table 1

supplementary figure legends
