# Supplementary figures and images for: Loss of ATF3 exacerbates liver damage through the activation of mTOR/p70S6K/ HIF-1α signaling pathway in liver inflammatory injury
Source: Cell Death Dis. 2018 Sep 5;9(9):910. doi: 10.1038/s41419-018-0894-1 (PMC6125320; doi:10.1038/s41419-018-0894-1)

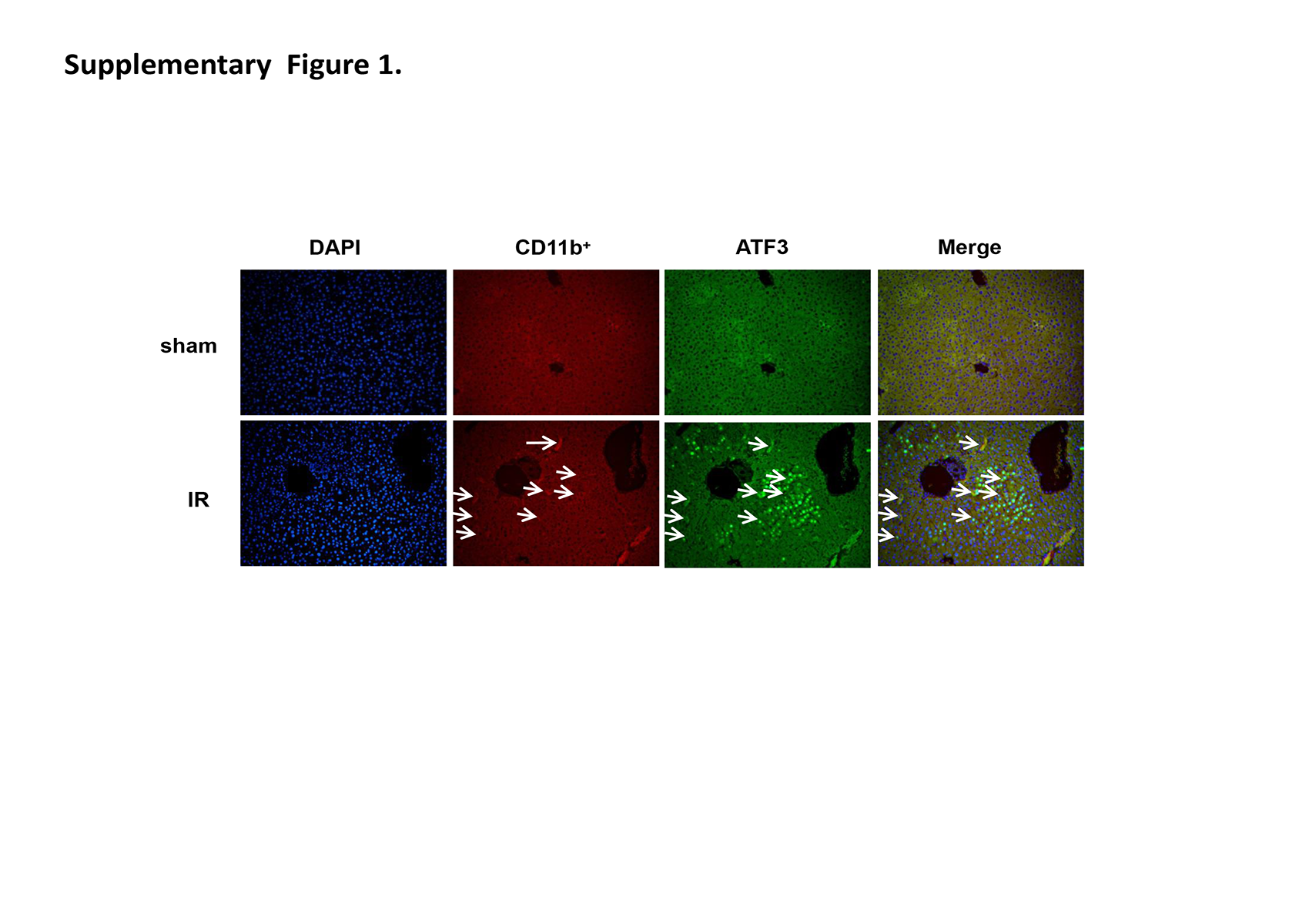

Supplement: Supplementary file 1 — Supplementary Figure 1 [file 41419_2018_894_MOESM1_ESM.tif]
