# Supplementary material for: Loss of ATF3 exacerbates liver damage through the activation of mTOR/p70S6K/ HIF-1α signaling pathway in liver inflammatory injury
Source: Cell Death Dis. 2018 Sep 5;9(9):910. doi: 10.1038/s41419-018-0894-1 (PMC6125320; doi:10.1038/s41419-018-0894-1)
Supplement: Supplementary file 2 — Supplementary Table 1 [file 41419_2018_894_MOESM2_ESM.docx]

| **Supplementary Table 1: Primer sequences for the amplification** | | |
| --- | --- | --- |
| Target genes | Forward primers | Reverse primers |
| HPRT | 5’-TCAACGGGGGACATAAAAGT-3’ | 5’-TGCATTGTTTTACCAGTGTCAA-3’ |
| TNF-α | 5’-ACGGCATGGATCTCAAAGAC-3’ | 5’-AGATAGCAAATCGGCTGACG-3’ |
| Il-6 | 5’-CTCTGGGAAATCGTGGAAATG-3’ | 5’-AAGTGCATCATCGTTGTTCATACA-3’ |
| IL-17A | 5’-TGTCCACCATGTGGCCTAAGAG-3’ | 5’-GTCCGAAATGAGGCTGTCTTTGA-3’ |
| IL-1β  ROR-γt  TGF-β | 5’-GCGGCCAGGATATAACTGACTTC -3’  5’-CATCTCCAGCCTCAGCTTTGA-3’  5’-TGCGCTTGCAGAGATTAAAA -3’ | 5’-GCGGCCAGGATATAACTGACTTC-3’  5’-CATCTCCAGCCTCAGCTTTGA-3’ 5’-CTGCCGTACAACTCCAGT -3’ |
