# Supplementary material for: Loss of ATF3 exacerbates liver damage through the activation of mTOR/p70S6K/ HIF-1α signaling pathway in liver inflammatory injury
Source: Cell Death Dis. 2018 Sep 5;9(9):910. doi: 10.1038/s41419-018-0894-1 (PMC6125320; doi:10.1038/s41419-018-0894-1)
Supplement: Supplementary file 3 — supplementary figure legends [file 41419_2018_894_MOESM3_ESM.docx]

**Supplementary Tables**

Table S1: The primer sequences used in qRT-PCR studies.

**Supplementary Figure1.**

**ATF3 expression was increased in IR-induced liver injury.**

Supplementary Figure1. Representative sections of liver immunostained for ATF3 and CD11b in Sham or IR groups. ATF3 signal was markedly detected in macrophages and hepatocyte from mouse livers subjected to 90min of warm ischemia followed by 6h of reperfusion.
